# UGLS: an uncertainty guided deep learning strategy for accurate image segmentation

**DOI:** 10.3389/fphys.2024.1362386

**Published:** 2024-04-08

**Authors:** Xiaoguo Yang, Yanyan Zheng, Chenyang Mei, Gaoqiang Jiang, Bihan Tian, Lei Wang

**Affiliations:** ^1^ Wenzhou People’s Hospital, The Third Affiliated Hospital of Shanghai University, Wenzhou, China; ^2^ School of Ophthalmology and Optometry, Eye Hospital, Wenzhou Medical University, Wenzhou, China

**Keywords:** deep learning, training strategy, image segmentation, fundus image, optic cup deep learning, optic cup

## Abstract

Accurate image segmentation plays a crucial role in computer vision and medical image analysis. In this study, we developed a novel uncertainty guided deep learning strategy (UGLS) to enhance the performance of an existing neural network (i.e., U-Net) in segmenting multiple objects of interest from images with varying modalities. In the developed UGLS, a boundary uncertainty map was introduced for each object based on its coarse segmentation (obtained by the U-Net) and then combined with input images for the fine segmentation of the objects. We validated the developed method by segmenting optic cup (OC) regions from color fundus images and left and right lung regions from Xray images. Experiments on public fundus and Xray image datasets showed that the developed method achieved a average Dice Score (DS) of 0.8791 and a sensitivity (SEN) of 0.8858 for the OC segmentation, and 0.9605, 0.9607, 0.9621, and 0.9668 for the left and right lung segmentation, respectively. Our method significantly improved the segmentation performance of the U-Net, making it comparable or superior to five sophisticated networks (i.e., AU-Net, BiO-Net, AS-Net, Swin-Unet, and TransUNet).

## 1 Introduction

Image segmentation is an important research direction of computer vision and medical image analysis, and widely used as a preprocessing step for various object detection and disease diagnosis (Khened et al., 2018; Jun et al., 2020). It can divide an image into several disjoint regions by performing a pixel-level classification and largely simplify the assessment of morphological and positional characteristics of object regions (Wang L. et al., 2022; Li et al., 2022). To accurately segment images, a number of image segmentation algorithms have been developed for many different applications, such as threshold based methods (Pare et al., 2019; Shahamat and Saniee Abadeh, 2020), active contour based methods (Han and Graphics, 2006), and random field based methods (Poggi and Ragozini, 1999; Hossain and Reza, 2017). Among these methods, deep learning based methods (Ronneberger et al., 2015; Wang Y. et al., 2022) have gained considerable popularity in the past decade because they can obtain remarkable segmentation performances comparable to manual annotations. Moreover, they are able to automatically extract and flexibly integrate different types of feature information by learning the intrinsic laws and representation levels of images to be segmented.

Despite promising performances, deep learning based methods are often faced with two key challenges in image segmentation (Wang et al., 2021c; Zheng et al., 2022), one is how to obtain rich local information, the other is how to robustly extract high-level semantics. Given the large number of parameters in deep learning networks, the spatial resolution of images generally decreases with the increase of network depth in order to speed up the learning of feature information. This resolution decrease can bring about the loss of local information, but the increase of network depth is beneficial to the acquisition of global semantic and context information. To mitigate these two challenges, different deep learning networks (Gawlikowski et al., 2023; Seoni et al., 2023) have been constantly emerging to accurately segment images with varying modalities. Alom et al. (Alom et al., 2019) put forward the RU-Net and R2U-Net, respectively by adding different cyclic convolutional blocks to the U-Net for feature detection and accumulation. Seo et al. (Seo et al., 2020) proposed a mU-Net model by introducing learnable deconvolution network structures into the U-Net to improve its learning ability at different resolutions and image segmentation performance. Huang et al. (Huang et al., 2020) proposed a U-Net 3+ model that combines high-level semantics with low-level semantics using full-scale jump concatenation to overcome the drawbacks of the U-Net and U-Net++ (Zhou et al., 2018). Cao et al. (Cao et al., 2022) and Chen et al. (Chen et al., 2021) proposed different transformer based networks (*i.e.*, Swin-Unet and TransUNet), respectively for accurate image segmentation. These network models demonstrated reasonable segmentation accuracy as compared to the U-Net, but their network structures were often more complex. This may not be conducive to network construction and training as well as image segmentation.

To avoid the design of complex network structures, we develop an uncertainty guided deep learning strategy (UGLS) in this study based on a existing network (*i.e.*, U-Net) for accurate image segmentation. We first train the U-Net to obtain a coarse segmentation result and then use morphological operations and Gaussian filters to identify a potential boundary region for each target object based on the obtained result. The boundary region has a unique intensity distribution to indicate the probability of each pixel belonging to object boundaries and is termed as the boundary uncertainty map (BUM) of the objects. With boundary uncertainty maps and original input images, we retrain the U-Net for the fine segmentation of target objects and can obtain a better performance, as compared to its coarse segmentation performance.

## 2 Methods

### 2.1 Scheme overview

**FIGURE 1:**
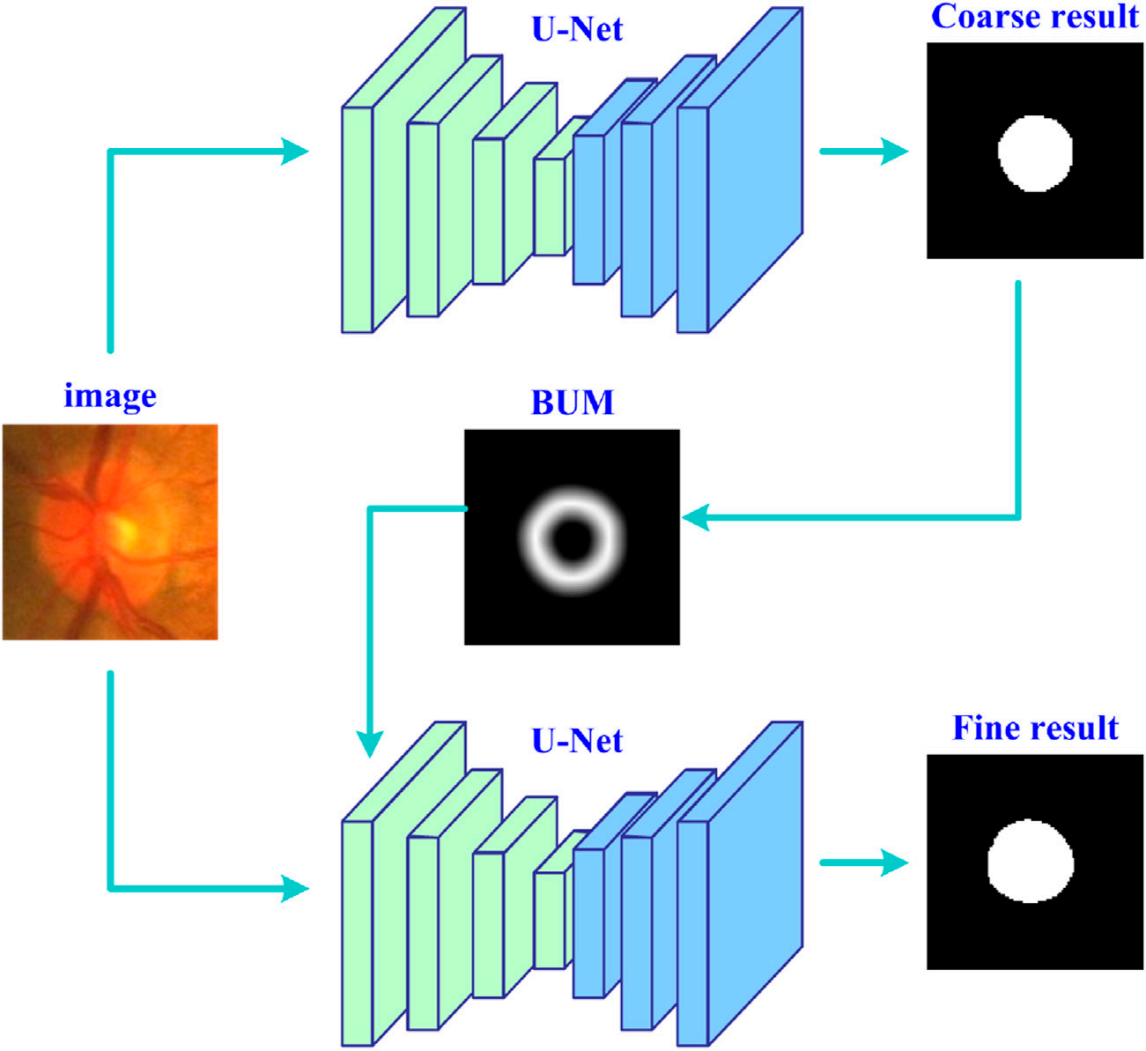
The flowchart of the developed deep learning strategy based on the U-Net for accurate image segmentation.

Figure 1 shows the entire workflow of the developed deep learning strategy (UGLS) based on a available network (*i.e*., U-Net) for image segmentation purposes. The UGLS consists of three key steps, namely, the coarse segmentation of target objects, generation of boundary uncertainty maps for each object, and object fine segmentation. The coarse segmentation is used to detect potential object regions and exclude irrelevant background far away from the detected regions. With the coarse segmentation, we can identify the regions where object boundaries are likely to appear and then generate boundary uncertainty maps for these objects, which can largely enhance the information about object boundaries and facilitate the boundary detection. We integrate these uncertainty maps and original input images and feed them into the given network for a more fine segmentation. After performing these three steps, the network can obtain a significantly improved segmentation performance.

### 2.2 Object coarse segmentation

We first trained the U-Net based on the given images and their manual annotations leveraging a plain network training scheme to obtain a relatively coarse segmentation result for desirable objects. This train procedure can be given by:

$$P=f\left(I,\varphi \right)$$ (1)

where I and P indicate the input image and its corresponding prediction map, respectively, $f\left(\cdot \right)$ denotes the U-Net with the network parameter φ . The prediction map was relatively coarse as compared with manual annotations of objects because the U-Net has a simple network structure and thereby limited potential to handle images with varying qualities.

### 2.3 Boundary uncertainty map

The obtained coarse segmentation results were often different from manual annotations of objects in certain image regions, especially object boundary regions, but they can provide some important position information for desirable objects. To effectively use the position information, we processed the coarse segmentation results leveraging morphological dilation and erosion operations (Fang et al., 2021), leading to two different object regions. Based on the two object regions, we can identify a potential boundary region (PBR) and a background excluded image (BEI) for each target object, which were separately given by

$$PBR=dilation\left(P,SE_{r}\right)-erosion\left(P,SE_{r}\right)$$ (2)

**FIGURE 2:**
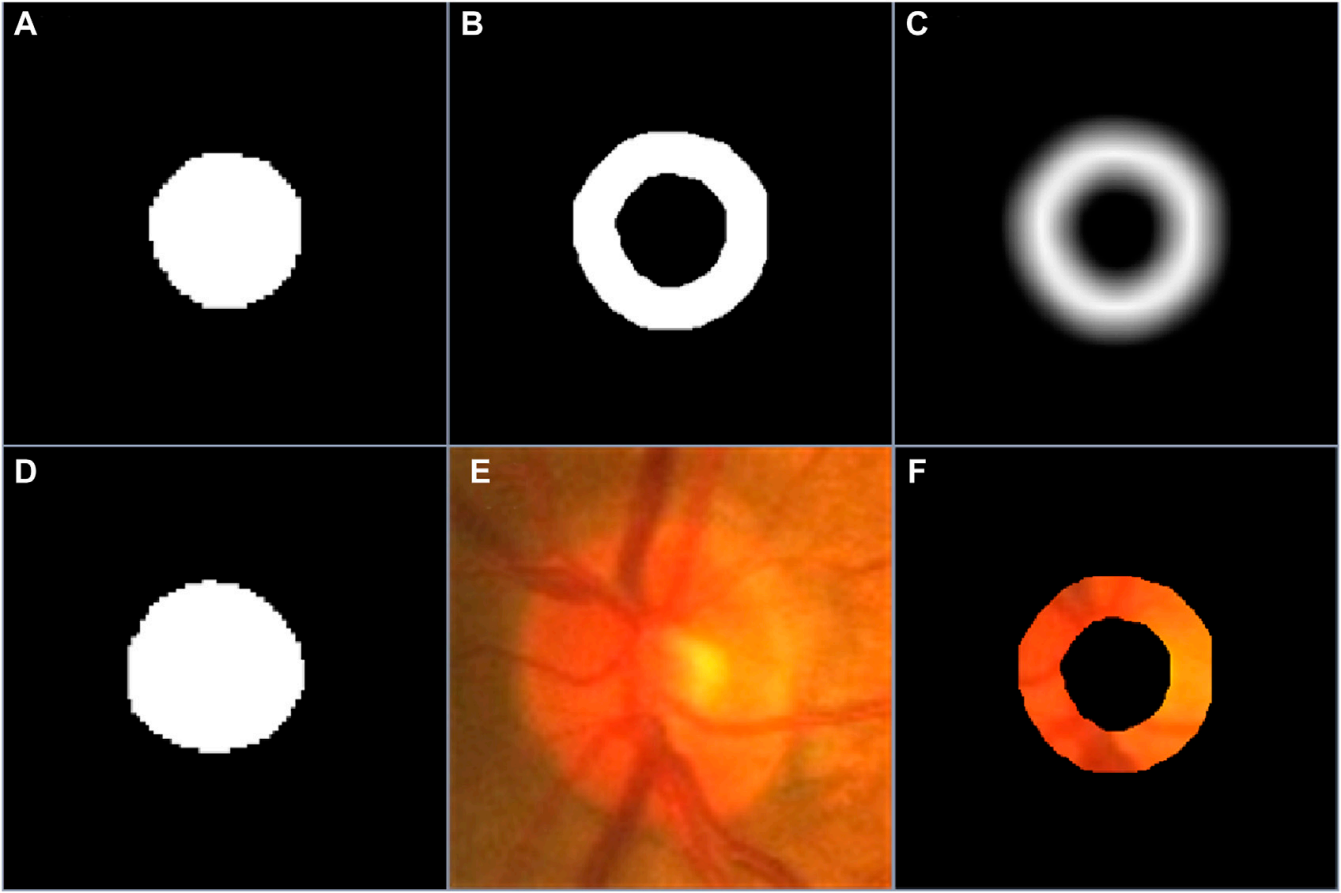
**(A–C)** are the coarse segmentation result, the PBR and boundary uncertainty map, respectively, **(D–F)** are the manual annotation of desirable object, the original image and its background excluded version.

BEI=PBR×I (3)

where $dilation\left(\cdot \right)$ and $erosion\left(\cdot \right)$ are the morphological dilation and erosion operations, respectively, SE is a circular structuring element with a radius of r . The PBR is a binary image and marks the region where object boundaries are most likely to appear, while the BEI merely retains the original image information located in the PBR and can reduce the impact of redundant background in image segmentation, as shown in Figure 2. To take fully advantage of edge position information in coarse segmentation results, we smoothed the PBR using a Gaussian filter with a rectangle window of r×r and a standard deviation of r to generate a boundary uncertainty map. The pixels in the uncertainty map took larger values when they were close to the center of the PBR and reduced ones when far away from this center. Moreover, A larger value generally means a higher probability that a pixel in the uncertainty map belongs to object boundaries. The unique intensity distribution made the boundary uncertainty map able to provide more relevant position information about object boundaries, as compared to the PBR.

### 2.4 Object fine segmentation

After obtaining the boundary uncertainty map and background excluded image, we concatenated these two types of images and fed them into the segmentation network. Since the concatenated images were different from the original images and contained very little background information, the segmentation network can easily detect object boundaries and thereby extract the whole object regions accurately using a simple experiment configuration. Specifically, we implemented the fine segmentation of desirable objects using the same configuration as their coarse segmentation (*e.g*., the cost function, optimizer and batch size).

### 2.5 Experiment datasets

**FIGURE 3:**
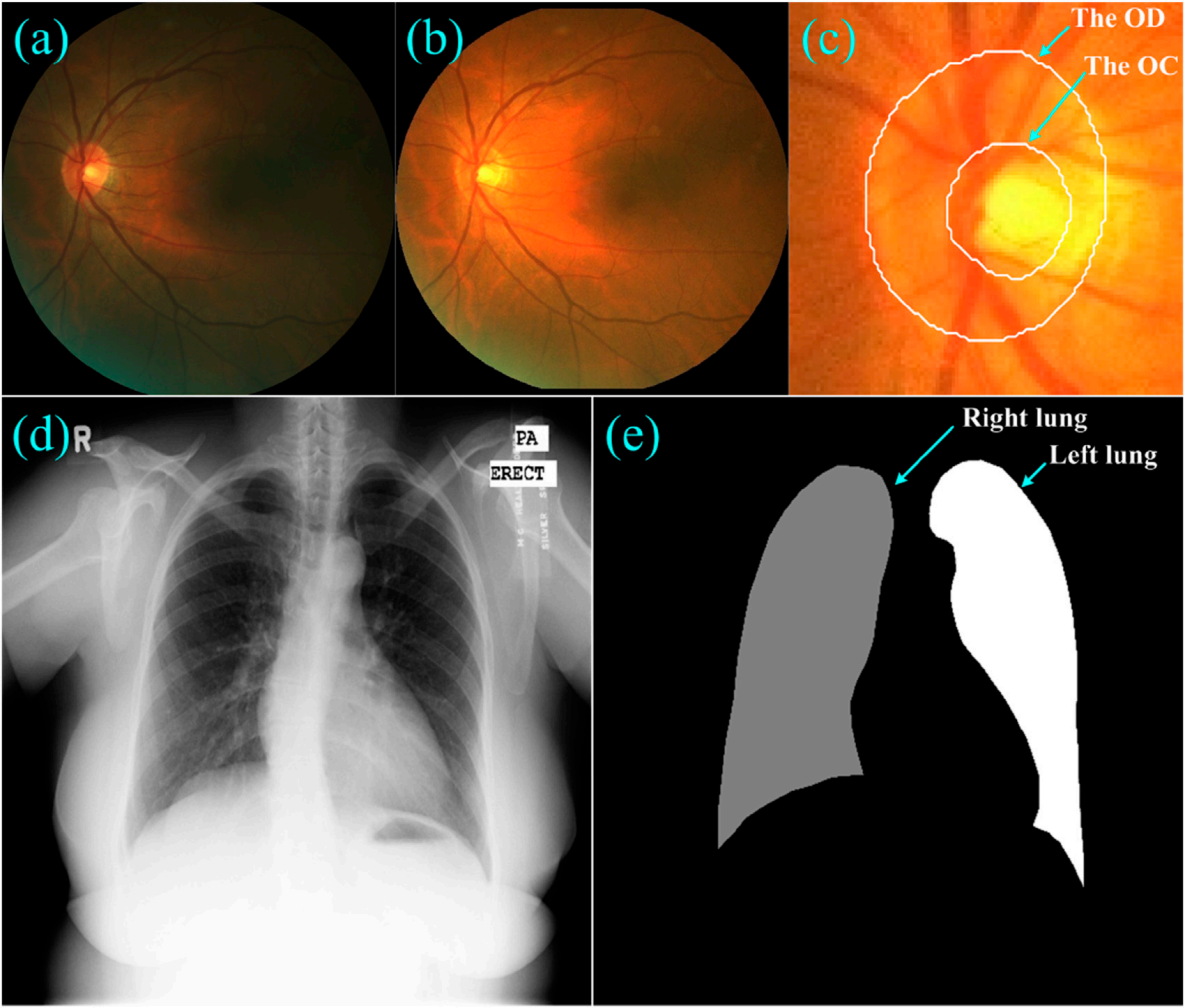
**(A–C)** showed a fundus image, its normalized version, and the local disc patch with manual annotations of the OD and OC, respectively, **(D)** and **(E)** showed a Xray image and its annotations for the left and right lungs.

To validate the developed learning strategy, we performed a series of segmentation experiments on two public dataset, as shown in Figure 3. The first dataset was from the Retinal Fundus Glaucoma Challenge (REFUGE) (Orlando et al., 2020) and contained 1,200 retinal fundus images acquired by two different cameras, together with manual annotations for the optic disc (OD) and cup (OC) regions. These images and their annotations were evenly split into three subsets for training (n = 400), validation (n = 400) and testing (n = 400) purposes, respectively, in the REFUGE challenge, which were also used in this study for segmentation purposes. We normalized these images to reduce the influence of light exposure and cameras and then extracted local disc patches using the dimensions that approximated three times the radius of the OD regions (Wang et al., 2021b). The extracted patches were then resized to 256 × 256 pixels and fed into the U-Net for network training.

The second dataset was from a tuberculosis screening program in Montgomery County (TSMC) (Jaeger et al., 2014) and contained 138 chest Xray images acquired using a Eureka stationary Xray machine. Among these Xray images, 80 were normal and 58 were abnormal with manifestations of tuberculosis. All images were de-identified and had a dimension of either 4,020 × 4,892 or 4,892 × 4,020 pixels. The left and right lungs depicted on these Xray images were manually annotated by a radiologist. We also split these Xray images equally into three disjoint subsets for network training (n = 46), validation (n = 46) and testing (n = 46), and resized them to the same dimension of 256 × 256 pixels.

### 2.6 Performance evaluation

We assessed the performance of the UGLS based on the U-Net (short for the developed method, https://github.com/wmuLei/ODsegmentation) on a 64-bit Windows 10 PC with 2.20 GHz 2.19 GHz Intel(R) Xeon(R) Gold 5120 CPU, 64 GB RAM and NVIDIA GeForce GTX 2080Ti by segmenting 1) the OC region from color fundus images and 2) the left and right lungs from the Xray images, where the r was set to 25 and 35, respectively for these two datasets. We used the Dice Score (DS) (Shi et al., 2022) as the cost function to assess the similarity between the segmentation results and their corresponding manual annotations for each object:

$$L=\frac{1}{K}\sum_{k=1}^{K}DS_{k}=\frac{1}{K}\sum_{k=1}^{K}\left(\frac{2\sum_{\Omega }p_{k,i}y_{k,i}}{\sum_{\Omega }p_{k,i}^{2}+\sum_{\Omega }y_{k,i}^{2}}\right)$$ (4)

where $DS_{k}$ denotes the DS for object k , and K is the total number of objects of interest. $p_{k,i}$ and $y_{k,i}$ are the output probabilities of a specific input image obtained by the U-Net and manual annotation, respectively for pixel i and object k , Ω denotes the entire image domain. We used the RMSprop optimizer to maximize the cost function and set its initial learning rate to 0.001, along with a batch size of eight and an epoch number of 100. To reduce the network training time, we halted the entire training procedure when the performance of the U-Net did not increase for 20 consecutive epochs. In addition, we randomly augmented input images during network training using some transformations, such as horizontal/vertical flip, scaling from 0.9 to 1.1, translation by −10 to 10 percent per axis, rotation from −180 to 180 in degree, and shearing from −5 to 5 in degree. After training, we binarized the prediction map of the U-Net using a given threshold of 0.5 to obtain desirable output results.

With these output results, we evaluated our developed method using the DS, Matthew’s correlation coefficient (MCC) (Zhu, 2020), sensitivity (SEN) (Wang et al., 2019), and Hausdorff distance (HSD, in pixel).

$$MCC=\frac{\left(TpTn-FpFn\right)}{\sqrt{\left(Tp+Fp\right)\left(Tp+Fn\right)\left(Tn+Fp\right)\left(Tn+Fn\right)}}$$ (5)

$$SEN=\frac{Tp}{Tp+Fn}$$ (6)

$$\text{HSD}=\max\left(d\left(X,Y\right),d\left(Y,X\right)\right)$$ (7)

where $T_{p}$ , $F_{p}$ , $T_{n}$ and $F_{n}$ denote the true positive, false positive, true negative and false negative, respectively. $d\left(X,Y\right)=\max_{x\in X}\min_{y\in Y}\left|x-y\right|$ is the directed HSD from point set X to Y . The larger the DS, MCC and SEN are and the smaller the HSD is, the better the segmentation performance of the network is. To show the advantage of the UGLS, we compared the developed method with the Attention U-Net (AU-Net) (Oktay et al., 2018), BiO-Net (Xiang et al., 2020), asymmetric U-Net (AS-Net) (Wang et al., 2021b), Swin-Unet (in tiny scale version), and TransUNet. Among these networks, U-Net and its variants (i.e., AU-Net, BiO-Net, AS-Net) shared the similar network architecture (e.g., the number of convolution filters increased from 32 to 1,024) and were trained from scratch based on a given dimension of 256 × 256 pixels and a learning rate of 0.001, while Swin-Unet and TransUNet were trained from initial ImageNet weights based on a dimension of 224 × 224 pixels and a learning rate of 0.01. All these networks were trained six times (by randomly arranging three different subsets for network training, validation and testing, respectively) using the same configurations (except for image dimension and learning rate) for each dataset. The paired *t*-test was used to evaluate the differences among the involved networks on the DS metric. A *p*-value less than 0.05 was considered statistically significant (Wang et al., 2021a).

## 3 Results

### 3.1 Object coarse segmentation

Tables 1 and 2 summarized six coarse segmentation results of the U-Net with the developed UGLS strategy in extracting the OC from retinal fundus images and the left and right lungs from Xray images, respectively. As demonstrated by the results, the U-Net achieved a relatively low performance in segmenting the OC depicted on fundus images (due to the high similarity between the OD and OC regions), with a average DS, MCC, SEN and HSD of 0.8642, 0.8585, 0.8674 and 2.6420, respectively. In contrast, it obtained a better accuracy for the left and right lungs (with the average DS of 0.9408 and 0.9477, respectively) and can compete with their manual annotations.

**TABLE 1:** Results of our proposed method for the coarse segmentation of the OC regions based on six experiments (i.e., Seg1-6) in terms of the mean and standard deviation (SD) of DS, MCC, SEN and HSD (in pixel).

Object	Result	DS	MCC	SEN	HSD
		Mean ± SD	Mean ± SD	Mean ± SD	Mean ± SD
OC	Seg1	0.8687 ± 0.0636	0.8629 ± 0.0627	0.8654 ± 0.1076	2.6266 ± 0.6213
	Seg2	0.8439 ± 0.0767	0.8380 ± 0.0749	0.8592 ± 0.1363	2.7302 ± 0.6845
	Seg3	0.8784 ± 0.0579	0.8720 ± 0.0563	0.9344 ± 0.0856	2.5711 ± 0.5217
	Seg4	0.8646 ± 0.0769	0.8586 ± 0.0757	0.8508 ± 0.1239	2.5952 ± 0.6782
	Seg5	0.8399 ± 0.0886	0.8358 ± 0.0847	0.7980 ± 0.1413	2.7719 ± 0.7082
	Seg6	0.8898 ± 0.0595	0.8839 ± 0.0590	0.8969 ± 0.0861	2.4492 ± 0.5737
	Overall	0.8642 ± 0.0736	0.8585 ± 0.0717	0.8674 ± 0.1230	2.6240 ± 0.6435

**TABLE 2:** The performance of the developed method for segmenting the left and right lungs (LL and RL) from Xray images.

Object	Result	DS	MCC	SEN	HSD
		Mean ± SD	Mean ± SD	Mean ± SD	Mean ± SD
LL	Seg1	0.9763 ± 0.0197	0.9733 ± 0.0216	0.9672 ± 0.0369	3.9530 ± 1.5336
	Seg2	0.9551 ± 0.0401	0.9512 ± 0.0399	0.9258 ± 0.0675	4.3733 ± 1.0238
	Seg3	0.9019 ± 0.1429	0.8938 ± 0.1520	0.9258 ± 0.0899	5.3338 ± 2.3926
	Seg4	0.9546 ± 0.0924	0.9521 ± 0.0906	0.9569 ± 0.0168	4.2231 ± 2.0033
	Seg5	0.9540 ± 0.0870	0.9518 ± 0.0798	0.9838 ± 0.0135	4.1766 ± 1.8032
	Seg6	0.9028 ± 0.0846	0.8977 ± 0.0789	0.9899 ± 0.0197	5.9666 ± 1.5560
	Overall	0.9408 ± 0.0917	0.9367 ± 0.0925	0.9582 ± 0.0558	4.6711 ± 1.9139
**Object**	**Result**	**DS**	**MCC**	**SEN**	**HSD**
		**Mean ± SD**	**Mean ± SD**	**Mean ± SD**	**Mean ± SD**
RL	Seg1	0.9656 ± 0.0418	0.9616 ± 0.0433	0.9826 ± 0.0500	4.6004 ± 1.6841
	Seg2	0.9519 ± 0.0464	0.9471 ± 0.0464	0.9242 ± 0.0787	5.0881 ± 1.1806
	Seg3	0.8962 ± 0.1268	0.8807 ± 0.1519	0.8991 ± 0.0567	6.6095 ± 2.9507
	Seg4	0.9552 ± 0.0893	0.9512 ± 0.0920	0.9537 ± 0.0181	5.0575 ± 2.2573
	Seg5	0.9644 ± 0.0899	0.9613 ± 0.0896	0.9784 ± 0.0407	4.6336 ± 1.5349
	Seg6	0.9531 ± 0.0707	0.9496 ± 0.0687	0.9655 ± 0.0543	4.7648 ± 1.7171
	Overall	0.9477 ± 0.0860	0.9419 ± 0.0940	0.9506 ± 0.0608	5.1257 ± 2.0893

### 3.2 Object fine segmentation

Tables 3 and 4 demonstrated the fine segmentation results of the U-Net with the developed UGLS strategy for three different objects depicted on fundus and Xray images, respectively. The U-Net achieved the average DS and SEN of 0.8791 and 0.8858 for the OC region, and 0.9605, 0.9607, 0.9621, and 0.9668 for the left and righ lungs, respectively. As compared with its coarse segmentation results, the U-Net obtained a significantly better overall performance for six different experiments on two types of images with varying modalities (*p* < 0.01). Specifically, the U-Net had better performances for five fine segmentation experiments for the OC, as compared to its coarse results, as shown in Table 3. Similarly, its performances were also increased in large increments for each experiment in the fine segmentation of the left and right lungs.

**TABLE 3:** Fine segmentation results of the developed method for the OC regions in terms of the DS, MCC, SEN and HSD (in pixel) metrics.

Object	Result	DS	MCC	SEN	HSD
		Mean ± SD	Mean ± SD	Mean ± SD	Mean ± SD
OC	Seg1	0.8696 ± 0.0647	0.8638 ± 0.0633	0.8804 ± 0.1036	2.5887 ± 0.6049
	Seg2	0.8442 ± 0.0755	0.8384 ± 0.0735	0.8764 ± 0.1306	2.7324 ± 0.6512
	Seg3	0.8691 ± 0.0661	0.8623 ± 0.0661	0.8835 ± 0.1147	2.5945 ± 0.6272
	Seg4	0.8982 ± 0.0547	0.8938 ± 0.0521	0.9141 ± 0.1027	2.2824 ± 0.4505
	Seg5	0.9033 ± 0.0527	0.8993 ± 0.0509	0.8573 ± 0.0922	2.2555 ± 0.5148
	Seg6	0.8904 ± 0.0612	0.8843 ± 0.0608	0.9033 ± 0.0821	2.4461 ± 0.5930
	Overall	0.8791 ± 0.0662	0.8737 ± 0.0651	0.8858 ± 0.1071	2.4833 ± 0.6031

**TABLE 4:** Fine segmentation results of the developed method for segmenting the left and right lungs (LL and RL) from the Xray images in terms of the DS, MCC, SEN and HSD (in pixel) metrics.

Object	Result	DS	MCC	SEN	HSD
		Mean ± SD	Mean ± SD	Mean ± SD	Mean ± SD
LL	Seg1	0.9764 ± 0.0206	0.9735 ± 0.0224	0.9709 ± 0.0349	3.9756 ± 1.4742
	Seg2	0.9636 ± 0.0405	0.9601 ± 0.0415	0.9552 ± 0.0612	4.0821 ± 0.9145
	Seg3	0.9291 ± 0.1325	0.9229 ± 0.1403	0.9148 ± 0.1511	4.6107 ± 1.8065
	Seg4	0.9619 ± 0.0940	0.9585 ± 0.1008	0.9675 ± 0.1007	3.6484 ± 1.5086
	Seg5	0.9680 ± 0.0412	0.9650 ± 0.0416	0.9794 ± 0.0182	3.9206 ± 1.2240
	Seg6	0.9639 ± 0.0565	0.9615 ± 0.0557	0.9762 ± 0.0341	4.1280 ± 1.7205
	Overall	0.9605 ± 0.0760	0.9569 ± 0.0800	0.9607 ± 0.0840	4.0609 ± 1.5006
**Object**	Result	DS	MCC	SEN	**HSD**
		Mean ± SD	Mean ± SD	Mean ± SD	**Mean ± SD**
RL	Seg1	0.9661 ± 0.0625	0.9630 ± 0.0616	0.9749 ± 0.0512	4.4255 ± 1.6023
	Seg2	0.9657 ± 0.0356	0.9618 ± 0.0365	0.9541 ± 0.0636	4.7426 ± 1.1453
	Seg3	0.9541 ± 0.0576	0.9480 ± 0.0636	0.9473 ± 0.0696	5.1637 ± 1.5636
	Seg4	0.9664 ± 0.0736	0.9625 ± 0.0809	0.9738 ± 0.0387	4.4790 ± 1.5192
	Seg5	0.9685 ± 0.0632	0.9653 ± 0.0654	0.9773 ± 0.0407	4.4856 ± 1.4704
	Seg6	0.9517 ± 0.0885	0.9491 ± 0.0850	0.9732 ± 0.0493	4.7916 ± 1.8466
	Overall	0.9621 ± 0.0658	0.9583 ± 0.0677	0.9668 ± 0.0546	4.6813 ± 1.5598

### 3.3 Performance comparison

**FIGURE 4:**
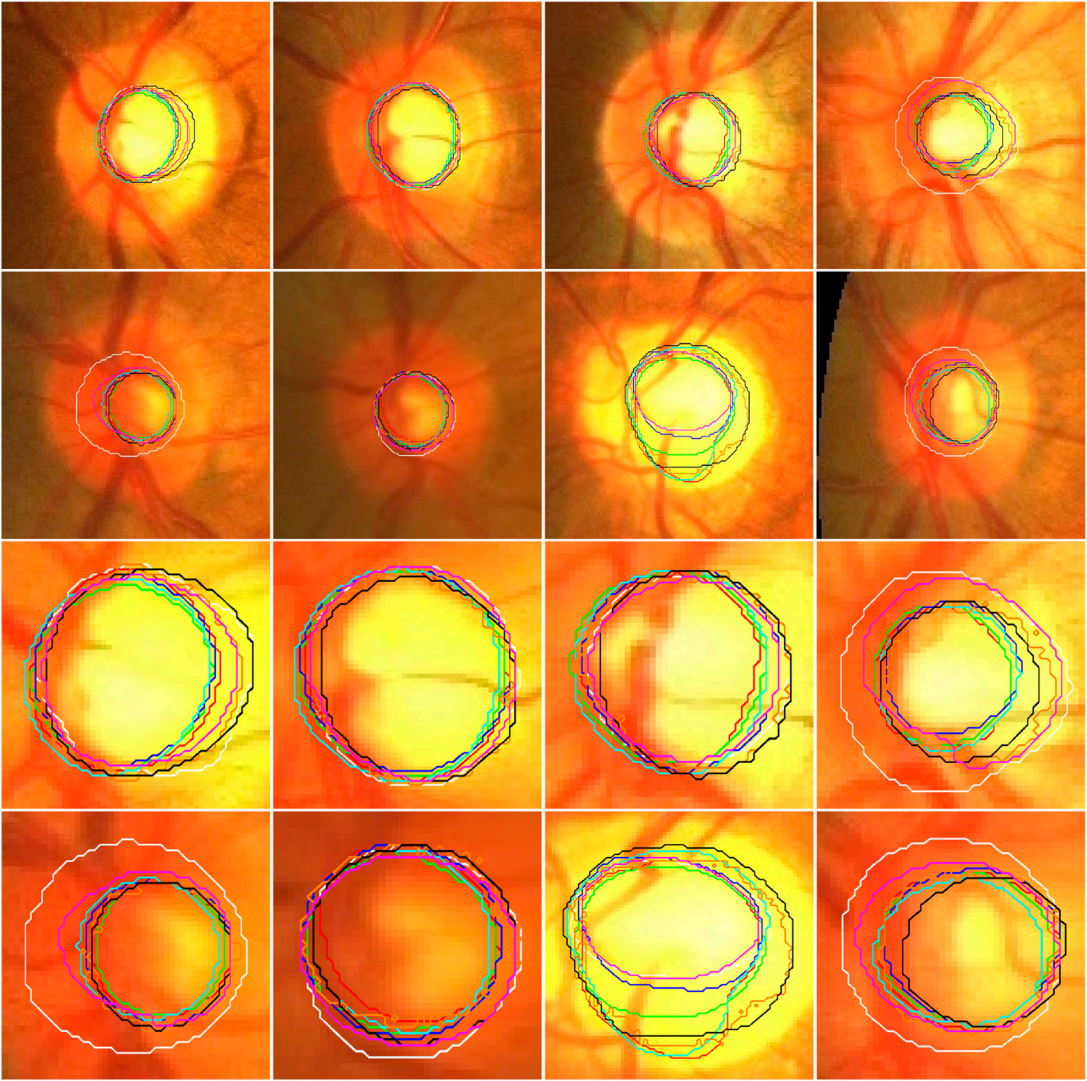
Illustration of the segmentation results of local disc patches (in the first two rows) and their closeup versions (in the last two rows) from eight fundus images obtained by the AU-Net (in green), BiO-Net (in blue), AS-Net (in cyan), Swin-Unet (in black), TransUNet (in orange) and our developed method in coarse (in red) and fine (in magenta) segmentation stages as well as their manual delineations (in white), respectively.

**FIGURE 5:**
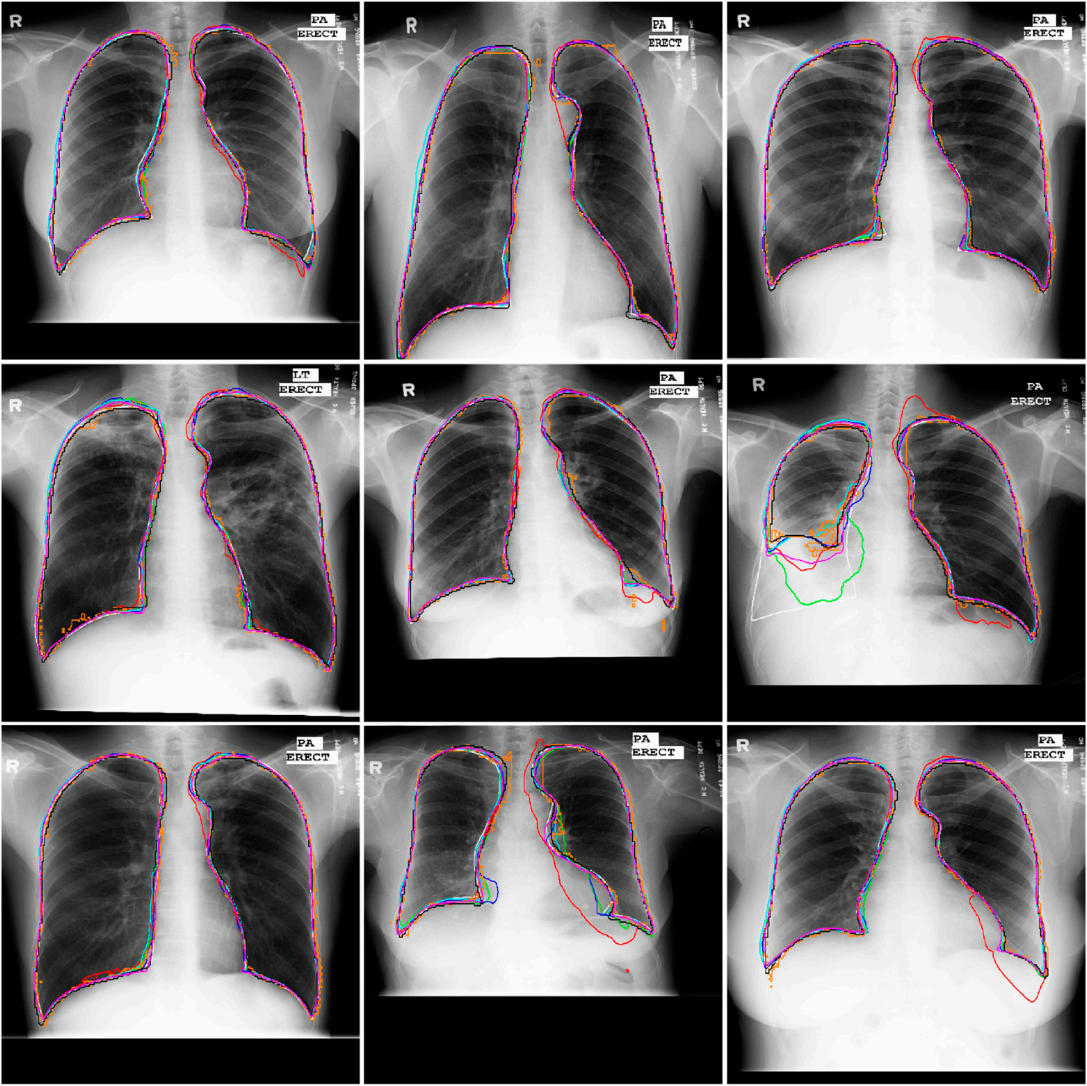
Illustration of the segmentation results of nine Xray images obtained by the AU-Net (in green), BiO-Net (in blue), AS-Net (in cyan), Swin-Unet (in black), TransUNet (in orange) and our developed method in coarse (in red) and fine (in magenta) segmentation stages as well as their manual delineations (in white), respectively.

Table 5 summarized the segmentation results of the involved networks (*i.e*., the U-Net, AU-Net, BiO-Net, AS-Net, Swin-Unet, and TransUNet) in extracting three different objects from fund and Xray images, respectively. As demonstrated by these results, the developed UGLS strategy can significantly improve the performance of the U-Net (*p* < 0.01) by merely leveraging the its coarse segmentation results in a reasonable way, instead of changing its network structure. Specifically, the average DS of the U-Net increased from 0.8792 to 0.8945 for three different object regions depicted on fundus and Xray images after using our developed deep learning strategy. This strategy made our developed method superior or comparable to the AU-Net (0.8803, *p* < 0.001), BiO-Net (0.8843, *p* < 0.005), AS-Net (0.8859, *p* < 0.005), Swin-Unet (0.8811, *p* < 0.001), and TransUNet (0.8900, *p* < 0.05) with all the *p*-values less than 0.05 for the two segmentation tasks. Figures 4 and 5 showed the performance differences among the involved networks on several fundus and Xray images.

**TABLE 5:** Performance differences among the involved networks in segmenting the OC, left and right lungs depicted on fundus and Xray images, respectively.

Object	Method	DS	MCC	SEN	HSD
		Mean ± SD	Mean ± SD	Mean ± SD	Mean ± SD
OC	U-Net	0.8642 ± 0.0736	0.8585 ± 0.0717	0.8674 ± 0.1230	2.6240 ± 0.6435
	AU-Net	0.8619 ± 0.0780	0.8569 ± 0.0746	0.8667 ± 0.1293	2.5896 ± 0.6180
	BiO-Net	0.8663 ± 0.0721	0.8605 ± 0.0704	0.8801 ± 0.1181	2.5960 ± 0.6374
	AS-Net	0.8676 ± 0.0725	0.8620 ± 0.0701	0.8757 ± 0.1186	2.6119 ± 0.6281
	Swin-Unet	0.8647 ± 0.0706	0.8582 ± 0.0689	0.8799 ± 0.1145	2.7134 ± 0.5767
	TransUNet	0.8737 ± 0.0643	0.8679 ± 0.0620	0.8894 ± 0.1071	2.4974 ± 0.5416
	Proposed	0.8791 ± 0.0662	0.8737 ± 0.0651	0.8858 ± 0.1071	2.4833 ± 0.6031
**Object**	**Method**	**DS**	**MCC**	**SEN**	**HSD**
		**Mean ± SD**	**Mean ± SD**	**Mean ± SD**	**Mean ± SD**
LL	U-Net	0.9408 ± 0.0917	0.9367 ± 0.0925	0.9582 ± 0.0558	4.6711 ± 1.9139
	AU-Net	0.9607 ± 0.0719	0.9572 ± 0.0758	0.9649 ± 0.0502	4.1158 ± 1.8849
	BiO-Net	0.9614 ± 0.0681	0.9582 ± 0.0673	0.9637 ± 0.0543	4.1554 ± 1.6769
	AS-Net	0.9649 ± 0.0493	0.9609 ± 0.0506	0.9623 ± 0.0633	4.7912 ± 1.6847
	Swin-Unet	0.9502 ± 0.0316	0.9446 ± 0.0317	0.9530 ± 0.0479	5.0613 ± 0.7958
	TransUNet	0.9617 ± 0.0357	0.9574 ± 0.0377	0.9604 ± 0.0476	4.1025 ± 1.0577
	Proposed	0.9605 ± 0.0760	0.9569 ± 0.0800	0.9607 ± 0.0840	4.0609 ± 1.5006
**Object**	**Method**	**DS**	**MCC**	**SEN**	**HSD**
		**Mean ± SD**	**Mean ± SD**	**Mean ± SD**	**Mean ± SD**
RL	U-Net	0.9477 ± 0.0860	0.9419 ± 0.0940	0.9506 ± 0.0608	5.1257 ± 2.0893
	AU-Net	0.9595 ± 0.0736	0.9545 ± 0.0804	0.9674 ± 0.0444	4.7496 ± 1.8938
	BiO-Net	0.9637 ± 0.0651	0.9597 ± 0.0661	0.9693 ± 0.0483	4.7352 ± 1.6805
	AS-Net	0.9663 ± 0.0516	0.9628 ± 0.0552	0.9636 ± 0.0537	4.1806 ± 1.6053
	Swin-Unet	0.9549 ± 0.0280	0.9488 ± 0.0281	0.9562 ± 0.0457	5.6140 ± 0.8730
	TransUNet	0.9602 ± 0.0407	0.9552 ± 0.0404	0.9606 ± 0.0421	4.8674 ± 1.1996
	Proposed	0.9621 ± 0.0658	0.9583 ± 0.0677	0.9668 ± 0.0546	4.6813 ± 1.5598

### 3.4 Effect of the BUM

Table 6 showed the results of the developed method in extracting the left and right lungs from Xray images using boundary uncertainty maps in three different ways. As demonstrated by the results, our developed method obtained the lowest segmentation performance, with the average DS of 0.9437 when merely trained on boundary uncertainty maps, but it had increased performance when combining the uncertainty maps with the original images or their background excluded version for network training (with the average DS of 0.9611 and 0.9613). Moreover, the background excluded images can better improve the performance of our developed method since they reduced the impact of irrelevant background information away from desirable objects.

**TABLE 6:** The results of the developed method trained on the boundary uncertainty map (BUM) or its combination with the original image (ORI) or its background excluded version (BEI) for the left and right lung segmentation.

Object	Method	DS	MCC	SEN	HSD
		Mean ± SD	Mean ± SD	Mean ± SD	Mean ± SD
LL	BUM	0.9347 ± 0.1227	0.9297 ± 0.1245	0.9356 ± 0.1284	4.3859 ± 1.5356
	ORI + BUM	0.9587 ± 0.0920	0.9553 ± 0.0956	0.9603 ± 0.0936	4.0907 ± 1.6055
	BEI + BUM	0.9605 ± 0.0760	0.9569 ± 0.0800	0.9607 ± 0.0840	4.0609 ± 1.5006
**Object**	**Method**	**DS**	**MCC**	**SEN**	**HSD**
		**Mean ± SD**	**Mean ± SD**	**Mean ± SD**	**Mean ± SD**
RL	BUM	0.9527 ± 0.0743	0.9476 ± 0.0771	0.9517 ± 0.0819	5.0905 ± 1.5155
	ORI + BUM	0.9634 ± 0.0683	0.9597 ± 0.0708	0.9651 ± 0.0636	4.6374 ± 1.5137
	BEI + BUM	0.9621 ± 0.0658	0.9583 ± 0.0677	0.9668 ± 0.0546	4.6813 ± 1.5598

### 3.5 Effect of parameter r

**FIGURE 6:**
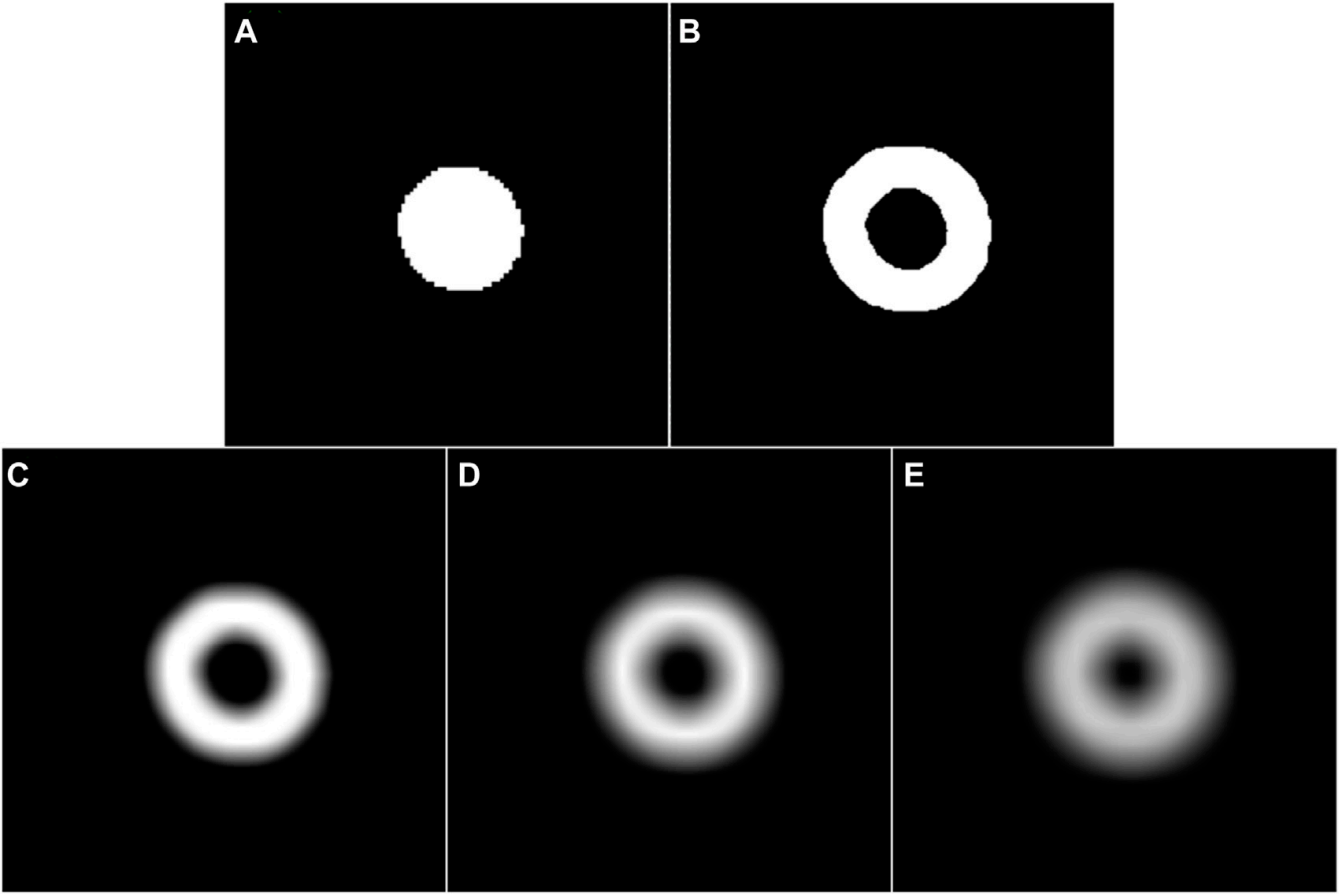
**(A)** and **(B)** are the coarse segmentation result of a given fundus image and its corresponding potential boundary region, respectively. **(C**–**E)** are the smoothed results of **(B)** using a Gassian filter with the parameter r of 15, 25, and 35, respectively.

Table 7 summarized the impact of the parameter r on the performance of the developed method in segmenting three different objects from fundus and Xray images. The developed method achieved the best overall performance when this parameter was set to 25 in the OC segmentation and 35 in the left and right lung segmentation, respectively, for the morphological operations and Gaussian filter. These two parameter values ensured a good balance between object information and irrelevant background for our developed method, making it able to accurately detect object boundaries. Table 8 showed the performance of the developed method when using different values for the parameters in the morphological operations and Gaussian filter. From the table, our developed method obtained a superior overall performance when the morphological operations and Gaussian filter shared the same value for each image dataset, which can effectively highlight the center regions of boundary uncertainty maps, as shown in Figure 6.

**TABLE 7:** The results of the developed method on fundus and Xray images by setting different values for parameters r .

Object	r	DS	MCC	SEN	HSD
		Mean ± SD	Mean ± SD	Mean ± SD	Mean ± SD
OC	15	0.8678 ± 0.0696	0.8626 ± 0.0680	0.8622 ± 0.1220	2.5426 ± 0.6246
	25	0.8791 ± 0.0662	0.8737 ± 0.0651	0.8858 ± 0.1071	2.4833 ± 0.6031
	35	0.8735 ± 0.0655	0.8679 ± 0.0639	0.8848 ± 0.1112	2.5257 ± 0.5995
**Object**	r	**DS**	**MCC**	**SEN**	**HSD**
		**Mean ± SD**	**Mean ± SD**	**Mean ± SD**	**Mean ± SD**
LL	15	0.9512 ± 0.1117	0.9474 ± 0.1173	0.9519 ± 0.1114	4.1688 ± 1.6956
	25	0.9543 ± 0.0968	0.9512 ± 0.0964	0.9566 ± 0.0987	4.1234 ± 1.6199
	35	0.9605 ± 0.0760	0.9569 ± 0.0800	0.9607 ± 0.0840	4.0609 ± 1.5006
	45	0.9566 ± 0.0996	0.9537 ± 0.0993	0.9582 ± 0.1017	4.1346 ± 1.5093
	55	0.9554 ± 0.1014	0.9523 ± 0.1034	0.9584 ± 0.1023	4.0513 ± 1.5453
**Object**	r	**DS**	**MCC**	**SEN**	**HSD**
		**Mean ± SD**	**Mean ± SD**	**Mean ± SD**	**Mean ± SD**
RL	15	0.9614 ± 0.0649	0.9578 ± 0.0643	0.9627 ± 0.0616	4.7130 ± 1.5723
	25	0.9617 ± 0.0713	0.9578 ± 0.0725	0.9646 ± 0.0529	4.6753 ± 1.6561
	35	0.9621 ± 0.0658	0.9583 ± 0.0677	0.9668 ± 0.0546	4.6813 ± 1.5598
	45	0.9625 ± 0.0683	0.9589 ± 0.0692	0.9662 ± 0.0558	4.6934 ± 1.5860
	55	0.9636 ± 0.0646	0.9602 ± 0.0641	0.9662 ± 0.0559	4.6227 ± 1.5216

**TABLE 8:** The results of the developed method for the first experiment on fundus and Xray images using different values for parameter r in morphological operations and Gaussian filter (short for $r_{m}$ and $r_{g}$ , respectively).

Object	( $r_{m}$ , $r_{g}$ )	DS	MCC	SEN	HSD
		Mean ± SD	Mean ± SD	Mean ± SD	Mean ± SD
**OC**	(25, 15)	0.8669 ± 0.0651	0.8611 ± 0.0639	0.8748 ± 0.1064	2.5936 ± 0.6169
	(25, 25)	0.8696 ± 0.0647	0.8638 ± 0.0633	0.8804 ± 0.1036	2.5887 ± 0.6049
	(25, 35)	0.8675 ± 0.0655	0.8619 ± 0.0642	0.8708 ± 0.1083	2.5815 ± 0.6131
**Object**	( $r_{m}$ , $r_{g}$ )	**DS**	**MCC**	**SEN**	**HSD**
		**Mean ± SD**	**Mean ± SD**	**Mean ± SD**	**Mean ± SD**
LL	(35, 15)	0.9695 ± 0.0333	0.9663 ± 0.0354	0.9679 ± 0.0459	4.2534 ± 1.7802
	(35, 25)	0.9733 ± 0.0259	0.9703 ± 0.0275	0.9702 ± 0.0378	4.1451 ± 1.7235
	(35, 35)	0.9764 ± 0.0206	0.9735 ± 0.0224	0.9709 ± 0.0349	3.9756 ± 1.4742
	(35, 45)	0.9750 ± 0.0262	0.9722 ± 0.0277	0.9752 ± 0.0357	3.9758 ± 1.5872
**Object**	( $r_{m}$ , $r_{g}$ )	**DS**	**MCC**	**SEN**	**HSD**
		**Mean ± SD**	**Mean ± SD**	**Mean ± SD**	**Mean ± SD**
RL	(35, 15)	0.9681 ± 0.0459	0.9646 ± 0.0465	0.9754 ± 0.0508	4.4583 ± 1.5158
	(35, 25)	0.9698 ± 0.0440	0.9666 ± 0.0446	0.9752 ± 0.0533	4.3794 ± 1.5588
	(35, 35)	0.9661 ± 0.0625	0.9630 ± 0.0616	0.9749 ± 0.0512	4.4255 ± 1.6023
	(35, 45)	0.9639 ± 0.0627	0.9610 ± 0.0608	0.9755 ± 0.0527	4.5328 ± 1.5872

## 4 Discussion

In this paper, we developed a novel network training strategy (termed UGLS) for accurate image segmentation and assessed its effectiveness based on an existing network (i.e., the U-Net) by extracting three different objects depicted (*i.e.*, the OC, left and right lungs) on fundus and Xray images. In the developed method, the U-Net was first trained using the traditional training strategy on the original images and their manual annotations for the coarse-grained segmentation of desirable objects. The segmentation results were then proposed to locate a potential boundary region for each object, which was combined with the original images for the fine segmentation of the objects. We validated the developed method on two public datasets (*i.e.*, REFUGE and TSMC) and compared it with five available networks (*i.e*., the AU-Net, BiO-Net, AS-Net, Swin-Unet and TransUNet) under the similar experiment configurations. Extensive experiments showed that the developed method can largely improve the segmentation performance of the U-Net and was comparable or superior to the AU-Net, BiO-Net, AS-Net, Swin-Unet and TransUNet, all of which had much more complex network structures than the U-Net.

The developed method achieved promising overall performance in segmenting multiple different objects, as compared to three existing networks. This may be attributed to the following reasons: First, the coarse segmentation of the objects was able to detect various types of image features and provide some important location information for each object and its boundaries. Second, the introduction of boundary uncertainty maps made the potential boundary region have a unique intensity distribution. This distribution largely facilitated the detection of object boundaries and enhanced the sensitivity and accuracy of the U-Net in segmenting objects of interest. Third, the use of background excluded images can not only ensure a reasonable balance between object information and its surrounding background, but also ensure that the U-Net performs the learning of various features in the specified region, thereby leading to a increased segmentation performance and a reduced influence of undesirable background. Due to these reasons, the developed method can significantly improve the segmentation performance of a relatively simple network (*i.e*., the U-Net) and make it comparable or superior to several existing sophisticated networks.

We further assessed the influence of boundary uncertainty maps and the parameter r on the performance of the developed method. Segmentation results in Tables 6–8 showed that (Eq. 1) the developed method achieved better segmentation performance when trained on the combination of boundary uncertainty maps and the background excluded images, as compared to the counterparts trained merely on boundary uncertainty maps or the original images. This may be due to the fact that there are no enough texture information relative to targe objects and their boundaries in boundary uncertainty maps, but too much background information in the original images, both of which can reduce the learning potential of the U-Net and deteriorate its segmentation performance. 2) The developed method obtained relatively high segmentation accuracy when the parameter r was assigned to 25 for the OC segmentation and 35 for the left and right lung segmentation. This parameter controlled the amount of information about desirable objects and their surrounding background in the boundary uncertainty maps. A proper value for the parameter can ensure a good balance between the two types of image information and significantly improve the fine segmentation performance of our developed method. If the parameter value was set too small or large, our developed method would have a final result that was very close to its coarse segmentation results or contained lots of undesirable background. 3) The parameter r was used simultaneously in morphological operations and Gaussian filter since it can ensure that pixels in the center region of boundary uncertainty map have more high contrast or intensity, as compared to the counterparts in other regions. 4) Boundary uncertainty maps can be generated using different strategies, but their corresponding segmentation performance was very similar (*i.e.*, 0.8791 vs. 0.8721 for the OC segmentation), based on our previous study (Zhang et al., 2023).

## 5 Conclusion

We developed a uncertainty guided deep learning strategy (UGLS) to improve the performance of existing segmentation neural networks and validated it based on the classical U-Net by segmenting the OC from color fundus images and the left and right lungs from Xray images. The novelty of our developed method lies in the introduction of boundary uncertainty maps and their integration with the input images for accurate image segmentation. Extensive experiments on public fundus and Xray image datasets demonstrated that the developed method had the potential to effectively extract the OC from fundus images and the left and right lungs from Xray images, largely improved the performance of the U-Net, and can compete with several sophisticated networks (*i.e*., the AU-Net, BiO-Net, AS-Net, Swin-Net, and TransUNet).

## Data Availability

Publicly available datasets were analyzed in this study. This data can be found here: Retinal Fundus Glaucoma Challenge (REFUGE), a tuberculosis screening program in Montgomery County.
